# Mass Mortality Events in the NW Adriatic Sea: Phase Shift from Slow- to Fast-Growing Organisms

**DOI:** 10.1371/journal.pone.0126689

**Published:** 2015-05-14

**Authors:** Cristina Gioia Di Camillo, Carlo Cerrano

**Affiliations:** Department of Life and Environmental Sciences, Polytechnic University of Marche, Ancona, Italy; Australian Institute of Marine Science, AUSTRALIA

## Abstract

Massive outbreaks are increasing all over the world, which are likely related to climate change. The North Adriatic Sea, a sub-basin of the Mediterranean Sea, is a shallow semi-closed sea receiving high nutrients inputs from important rivers. These inputs sustain the highest productive basin of the Mediterranean Sea. Moreover, this area shows a high number of endemisms probably due to the high diversity of environmental conditions and the conspicuous food availability. Here, we documented two massive mortalities (2009 and 2011) and the pattern of recovery of the affected biocoenoses in the next two years. Results show an impressive and fast shift of the benthic assemblage from a biocoenosis mainly composed of slow-growing and long-lived species to a biocoenosis dominated by fast-growing and short-lived species. The sponge *Chondrosia reniformis*, one of the key species of this assemblage, which had never been involved in previous massive mortality events in the Mediterranean Sea, reduced its coverage by 70%, and only few small specimens survived. All the damaged sponges, together with many associated organisms, were detached by rough-sea conditions, leaving large bare areas on the rocky wall. Almost three years after the disease, the survived specimens of *C*. *reniformis* did not increase significantly in size, while the bare areas were colonized by fast-growing species such as stoloniferans, hydrozoans, mussels, algae, serpulids and bryozoans. Cnidarians were more resilient than massive sponges since they quickly recovered in less than one month. In the study area, the last two outbreaks caused a reduction in the filtration efficiency of the local benthic assemblage by over 60%. The analysis of the times series of wave heights and temperature revealed that the conditions in summer 2011 were not so extreme as to justify severe mass mortality, suggesting the occurrence of other factors which triggered the disease. The long-term observations of a benthic assemblage in the NW Adriatic Sea allowed us to monitor its dynamics before, during and after the mortality event. The N Adriatic Sea responds quickly to climatic anomalies and other environmental stresses because of the reduced dimension of the basin. The long-term consequences of frequent mass mortality episodes in this area could promote the shift from biocoenoses dominated by slow-growing and long-lived species to assemblages dominated by plastic and short life cycle species.

## Introduction

In the last 30 years, the general warming trend, the alteration of the carbon and nitrogen cycles and the increase in the frequency of exceptional meteorological events triggered profound changes in the thermohaline circulation of the Mediterranean Sea, affecting the dynamics of the basin in both deep and shallow waters [1–3]. The Mediterranean Sea is a hotspot of biodiversity with a high rate of endemism [4–5], but the climate change and the high anthropogenic pressure are giving rise to important shifts in the biota. Frequent disease outbreaks, invasion of non indigenous species, increase in the abundance of thermophilic organisms and the regression of cold-affinity species, loss of foundation species [6] and shortening of reproductive periods are some of the drastic consequences of the environmental changes [3, 7–8].

In recent years several mass mortality episodes have occurred along the Western Mediterranean coasts affecting above all sponges and cnidarians [7, 9–15].

There is some evidences that global warming is leading to a general lengthening of summer conditions and water stratification with consequent exposure of benthic organisms to prolonged periods of high temperature and low food availability [7]. Hence, the mortality episodes occurring in late summer are strictly related to climatic anomalies [7]. However, other concomitant environmental stresses—such as mechanical injuries—probably compromise animal health and enhance their susceptibility to bacterial infections and diseases [16–17].

The North Adriatic Sea can be considered as the largest shelf area of the Mediterranean [18]. Due to its shallowness, the basin shows a temperate climate with very low winter temperature (about 7°C) and vertical stratification in summer [19–20]. The conspicuous fresh water inputs make the basin among the most productive of the Mediterranean [18]. Many of the outbreaks which occurred in the northern sector of the basin were due to its shallowness, high water temperature, low winds and stable sea conditions that drive water stratification, prevent pollutant dispersion and trigger hypoxic crisis [21–24].

The Adriatic Sea hosts 49% of the diversity of the Mediterranean [25]. In particular, the North Adriatic Sea shows a high number of endemisms probably due to the high peculiarity of its environmental conditions, such as its isolation and the conspicuous food availability for all trophic levels, from phytoplankton to fish [18, 26–27]. Here, benthic organisms reach considerable sizes or abundance [28–30] mainly due to high productivity rates comparable to those of the North Sea [31].

Historical data show the consequences of several human impacts on coastal waters worldwide, including the North Adriatic Sea [32]. Overfishing and destructive fishing methods, aquaculture, introduction of non indigenous species, eutrophication, pollution, tourism and population growth [27, 33]. are among the main threats in the Mediterranean and Adriatic seas, leading to the disturbance or destruction of natural habitats, alteration of their functioning and depletion of biodiversity [3, 25, 27], with cascade effects on the entire food web [32–34].

Several episodes of mass mortality have been reported even in the North Adriatic Sea during late summer [35], Table 1. In September 2009, an important sponge disease was reported from the western side of the central Adriatic Sea (Conero Promontory, Ancona). The event affected about 30% of the population of three dictyoceratids, *Ircina variabilis*, *Sarcotragus spinosulus*, and *Spongia officinalis* [35]. In autumn 2011, a new outbreak took place at the Conero Promontory involving a higher number of species and with more drastic consequences. The aim of this work is to analyze and quantify the effects of this last wide mortality event and to compare the abundance of the affected organisms before, during and after the disease. In particular, we monitored this site two and three years after the event in order to evaluate the resilience of the damaged assemblage.

**Table 1:** Abundance of some invertebrate species at Conero Promontory (NW Adriatic Sea) before, during and after the disease.

Species	Annual average abundance (data collected before the disease, from 2006 to 2010)	Average abundance in Septemebr and October (data collected before the disease, from 2006 to 2010)	Abundance during the disease (06 Oct 2011, present work)	Abundance after the disease (03 Nov 2011, present work)
*Eudendrium racemosum* (hydranths cm^-2^ of colony surface ± SD)	Annual average 32.3 ± 19.3 [28]	September 79.7 ± 24 October 26.7 ± 19 [28]	Colonies present but almost completely lacking of polyps	Fully recovered colonies with 34.1 hydranths cm^-2^ ± 4.8
*Epizoanthus arenaceus* (zoanthid cover (%± SD))	Annual average 13.2% ± 8.2 [30]	September 6.8% ± 10.9; October 12.5% ± 17 [30]	Most of the polyps were contracted 2.9%±1.7	Fully recovered polyps 8.2% ± 9.9
*Chondrosia reniformis* (sponge cover (%± SD))	Annual average 43.2% ± 6.8 [29]	September 43.8% ± 8.7 October 53.2% ± 0.5 [29]	63.1% ± 25.0 (damaged surface: 98.2% ± 4.2)	6.3% ± 4.9 (damaged surface: 0%)
*Tedania anhelans* (sponge cover (%± SD))	Annual average 4.9% ± 3.4 [29]	September 2.9% ± 2.2 October 1.6% ± 1.7 [29]	6.9% ± 3.8 (damaged surface: 0%)	2.9% ± 2.5 (damaged surface: 0%)
Irciniidae-Spongiidae (sponge cover (%± SD))	Average in June 2009 14.7% ± 18.3; (before the disease of 2009) (unpublished)	October 2009 (after disease) 15.3% ± 15.4 (unpublished)	19.7% ± 13.2 (damaged surface: 56.5% ± 46.1)	10.5% ± 8 (damaged surface of 9.6% ± 17.3)
Irciniidae-Spongiidae (sponge m^-2^ ± SD)	Average in June 2009 9.5 ± 16.5; (before the disease of 2009) [35]	October 2009 (after disease) 6.8 ± 1.1 [35]	5.7 ± 9.3	3.1 ± 9.8
*Cornularia cornucopiae* (thousands of polyps m^-2^ ± SD)	Annual average 12.7 ± 8.7 [36]	September 19.8 ± 1.9 October 8.6 ± 0.8 [36]	2.2 ± 3.5 (all the polyps were contracted)	Fully recovered colonies with 20.3 ± 16.6

## Materials and Methods

The Conero Promontory (North Adriatic Sea) was struck by a wide-spread disease in October 2011. The sampling site was the richest spot of the area in terms of biodiversity and with homogeneous characteristics such as depth range, light exposition, inclination and water movement (the northward side of the ‘Scoglio del Trave’, 43°34’54.05” N, 13°34’15.15” E). This site has been the object of several studies in the past, so there is a good general knowledge regarding several species living at the selected site (Table 1) (*Tedania* (*Tedania*) *anhelans* and *Chondrosia reniformis* [29], *Ircinia variabilis*, *Sarcotragus spinosulus* and *Spongia officinalis* [35], *Eudendrium racemosum* [28], *Cornularia cornucopiae* [36], *Epizoanthus arenaceus* [30]). These species can be considered as the most representative and abundant benthic invertebrates of the area.

### Environmental parameters

In order to hypothesize possible environmental causes of the disease, we considered the superficial sea-water temperature and wave height. The temperatures were downloaded from the National Tidegauge Network website [37]. Data of water temperature were daily collected from the transducer T020 TTA placed on the sea surface in the Ancona’s NT station (43° 37' 29.16''; 13° 30' 23.46'') each 10 minutes. Data of wave motion (wave height) of the Conero Promontory were collected by the Watchkeeper buoy of the Italian Data Buoy Network (RON) placed in 43°49'26’N; 13°43'10’E and supplied by the “Istituto Superiore per la Protezione e la Ricerca Ambientale ISPRA” (Rome) and the “Università Politecnica delle Marche UNIVPM” (Ancona).

The trends of weekly average values of wave height (m) and temperature (°C) of 2011 were calculated in order to highlight the sea and weather conditions around the period of the disease.

Since the majority of the disease occurs during or after a period of calm sea and high temperature [35], data of wave height and temperature were compared from 2000 to 2013. Moreover, we compared the temperature time series of the warmer period (from July to September) of 2010, 2011 and 2012 (present work) and 2007, 2008 and 2009 [35].

### Analysis of the impacts

In order to describe the effects of the mortality event on benthic assemblages, we evaluated the abundance of the above mentioned target species analysing underwater pictures taken before (2009 and 2010), during (6 October 2011), immediately after (03 November 2011), and two (June 2013) and three (June 2014) years after the mortality event. For each temporal interval, 40 pictures were taken haphazardly along a fixed 40 m long x 1 m wide horizontal transect at a depth of 5–7 m. A frame of 20 cm x 15 cm was used to take standardized pictures at intervals of 1 m along the transect and at a distance of 50 cm from the substrate using a Canon Ixus 960 IS digital underwater camera. In order to determine the abundance of the selected taxa, photographs were analysed by the ImageJ software [38].

Considering the different patterns of growth of the selected species, we evaluated their abundance as 1) percentage of covered substrate for *E*. *arenaceus*, *T*. *anhelans*, *C*. *reniformis* and Ircinidae-Spongidae complex, 2) density of specimens m^-2^ for *C*. *cornucopiae* and 3) number of polyps cm^-2^ of colony surface for *E*. *racemosum*.

Concerning the sponges, the abundance of the Irciniidae and Spongidae were considered together since it is difficult to distinguish *S*. *spinosulus* from *S*. *officinalis* underwater due to their similar morphology, while *I*. *variabilis* is quite rare. The sponge surfaces were determined without considering the thickness of the sponge body. Diseased sponges were easily recognized by white necrotic areas, hence, the collected pictures were analyzed to determine the percentage of damaged organisms and the percentage area of the necrotic tissue was evaluated for *C*. *reniformis*, *T*. *anhelans* and the Irciniidae-Spongidae complex.

The abundance of Irciniidae-Spongidae (specimens m^-2^) was also determined during the sampling activities of October and November 2011. The specimens present along the 40 m^2^ linear horizontal transect were counted underwater and were distinguished between healthy, partially and totally damaged animals. Hence, the percentage of each category was calculated.

Colonies of *Eudendrium racemosum* did not disappear during the disease but they lost their polyps. Hence, in order to evaluate the average hydranth density (hydranths cm^-2^ of colony surface ± SD) during and after the disease, ten colonies of the hydroid were randomly collected along the rocky wall at a depth of 5–7 m in October and November 2011; in the laboratory, samples were observed under a stereomicroscope to count the hydranths present in each collected colony. Later, the ImageJ software was used to measure the projected area of each colony and the number of hydranth per unit surface was determined.

### Recovery

In order to verify the resilience of the area, we compared the values of abundance (percentage of covered substrate ± SD) of macrobenthic organisms in June 2009, June 2013 and June 2014. We chose June since spring precedes mortality episodes generally occurring in late summer. Moreover, during summer, *Eudendrium racemosum* grows and becomes one the most abundant species, forming a dense belt of colonies along the wall [28]. The analyses were performed before the period of its maximum expansion in order to avoid the overestimation of the abundance of hydrozoan colonies.

Values of abundance were estimated using the same picture series taken in June 2009, 2013 and 2014 in order to analyze the impacts, but distinguishing between areas where *C*. *reniformis* was the dominant species (CrAs = *C*. *reniformis* Areas) and areas where the sponge did not predominate (OAs = Other Areas; depth). The taxa considered for the survey are: encrusting sponges (such as *Chondrosia reniformis*, *Tedania anhelans*), massive sponges (including *Dysidea avara*, *Ircinia variabilis*, *Sarcotragus spinosulus*, *Spongia officinalis*), cnidarians (*Eudendrium racemosum*, *Eudendrium merulum*, *Obelia dichotoma*, *Epizoanthus arenaceus*), *Mytilus galloprovincialis*, *Spirobranchus triqueter*, *Schizobrachiella sanguinea*, *Titanoderma* sp. and other less abundant organisms (*Rocellaria dubia*, *Pholas dactylus*, *Lithophaga lithophaga*, *Serpula vermicularis*, *Protula tubularia*, *Phallusia nigra*, *Microcosmus* sp., *Lima* sp., *Ostrea* spp., cirripeds, didemnids). We also took in account another conspicuous component of the benthic assemblages, represented by brownish mats (BM) mainly composed of cnidarians (the octocoral *Cornularia cornucopiae* and stolonal hydrozoans such as *Campanularia hincksii*, *Clytia gracilis*, *Bougainvillia* sp.), small tubeworms (syllids and *Sabellaria* sp.), tube-builder amphipods and sediment.

*C*. *reniformis* in the CrAs used to grow on or between some organisms such as the terebellid *Eupolymnia nebulosa*, the tunicates *Microcosmus* sp. and the polychaete *Serpula vermicularis*. In order to verify possible variation in the fauna associated to *C*. *reniformis*, the abundance (number of specimens m^-2^) of these suspension feeders was also determined before disease (June 2009), immediately after (November 2011) and two years later (June 2013).

We hypothesized that the abundance varied significantly over time and to validate our hypothesis, non-transformed data were tested by means of Kruskal-Wallis [39]. The non-parametric procedure was applied because data, checked with Shapiro-Wilk’s test, were not normally distributed. Analyses were performed using PAST for Windows version 1.91 [40].

*C*. *reniformis* and the Irciniidae-Spongiidae complex were particularly affected by the outbreak of 2011. *C*. *reniformis*, with its specimens larger than 1m^2^, was the largest sponge of the area and one of the most abundant benthic organisms [29]. In order to evaluate the variation of the cover (%) of *C*. *reniformis* and Irciniidae-Spongiidae complex affected by the two recent outbreaks, we compared pictures of a 0.5 x 0.5 m marked area taken in June 2009 (before the disease of 2009 [29], October 2009 (after the disease of 2009 [35], and after the disease of 2011 (November 2011 and June 2013) by the ImageJ software. The growth rate of the fragments of *C*. *reniformis* that survived during the disease of 2011 in the marked area, was estimated as changes in area over time [41] by analysing the pictures taken in November 2011 and June 2013. The rate was calculated considering the variation of the surface in 19 months without taking into account sponge thickness [42].

In order to estimate the possible loss in the filtration effectiveness of the communities living in the sampling area, the filtration rates reported in literature for some species (L h^-1^ ind^-1^; Table 2) and the abundance values (number of specimens m^-2^) of these animals at the studied site “Scoglio del Trave” were used to estimate the total filtered volume (L h^-1^ ind^-1^ m^-2^) before and after the diseases of 2009 and 2011. Considering that the sampling site is about 1 km long and 6 m deep, the rough total volume filtered by suspension feeders at Scoglio del Trave and its decrease from 2009 to 2013 were estimated.

**Table 2:** Filtration rates of some species from the study area before and after the disease of 2009 and almost two years after the disease of 2011.

Species	Estimated filtration rate	References	Abundance at Scoglio del Trave (n° ind. m^-2^)		Filtred volume (L h^-1^ m^-2^)		Abundance at Scoglio del Trave (n° ind. m^-2^)	Filtred volume (L h^-1^ m^-2^)	Total volume filtered along the north side of Scoglio del Trave (L h^-1^ in a 1000 m x 6 m area)			Reduction of the filtration power from 2009 to 2013	
			Before disease 2009	After disease 2009	Before disease 2009	After disease 2009	After disease 2011	After disease 2011	Before disease 2009	After disease 2009	After disease 2011	From June to Oct 2009	From Oct 2009 to June 2013
*Eupolymnia nebulosa*	7.5 10^–4^ (L h^-1^ ind^-1^)	[93]	43.7	probably unvaried	0.03	0.03	0	0	196.6	196.6	0	0%	100%
*Serpula vermicularis*	0.04 (L h^-1^ ind^-1^)	[94]	83.2	probably unvaried	3.3	3.3	71.6	2.9	20 10^3^	20 10^3^	17 10^3^	0%	14%
*Microcosmus* spp.	210 (L h^-1^ ind^-1^)	[95]	54.4	probably unvaried	11 10^3^	11 10^3^	32.6	7 10^3^	68 10^6^	68 10^6^	41 10^6^	0%	40%
*Phallusia* spp.	150 (L h^-1^ ind^-1^)	[96]	17.2	probably unvaried	2.6 10^3^	2.6 10^3^	3.8	570	15 10^6^	15 10^6^	3.4 10^6^	0%	78%
Irciniidae-Spongiidae	1 (L h^-1^ cm^-3^)	[97]	9.5 (with a volume* of 278 cm^3^)	6.8 (with a volume* of 193 cm^3^)	2.6 10^3^	1.3 10^3^	10.3 (with a volume* of 87 cm^3^)	900	16 10^6^	8 10^6^	5 10^6^	50%	16%
Chondrosia reniformis**	1 (L h-1 cm^-3^)	[98]	4 (with a volume* of 9828 cm^3^)	probably unvaried	9.8 10^3^	9.8 10^3^	25 (with a volume* of 12 cm^3^)	302	60 10^6^	60 10^6^	2 10^6^	0%	97%
								TOTAL	160 10^6^	150 10^6^	52 10^6^	5%	62%

*The sponge volume is calculated multiplying the average sponge surface (cm^2^) obtained by photo surveys by a thickness of 5 cm.

**Considered only big specimens of *C*. *reniformis*

### Ethics statement

No specific permits were required for the described field surveys. The field studies did not involve endangered or protected species. Our data are mainly based on not destructive sampling methods (photo surveys), the only one collected species was the hydroid *Eudendrium racemosum*, but no permits were necessary for this species and we preserved the integrity of the basal stolons (hydrorhizae) to allow the regeneration of the colonies.

## Results

**Fig 1:**
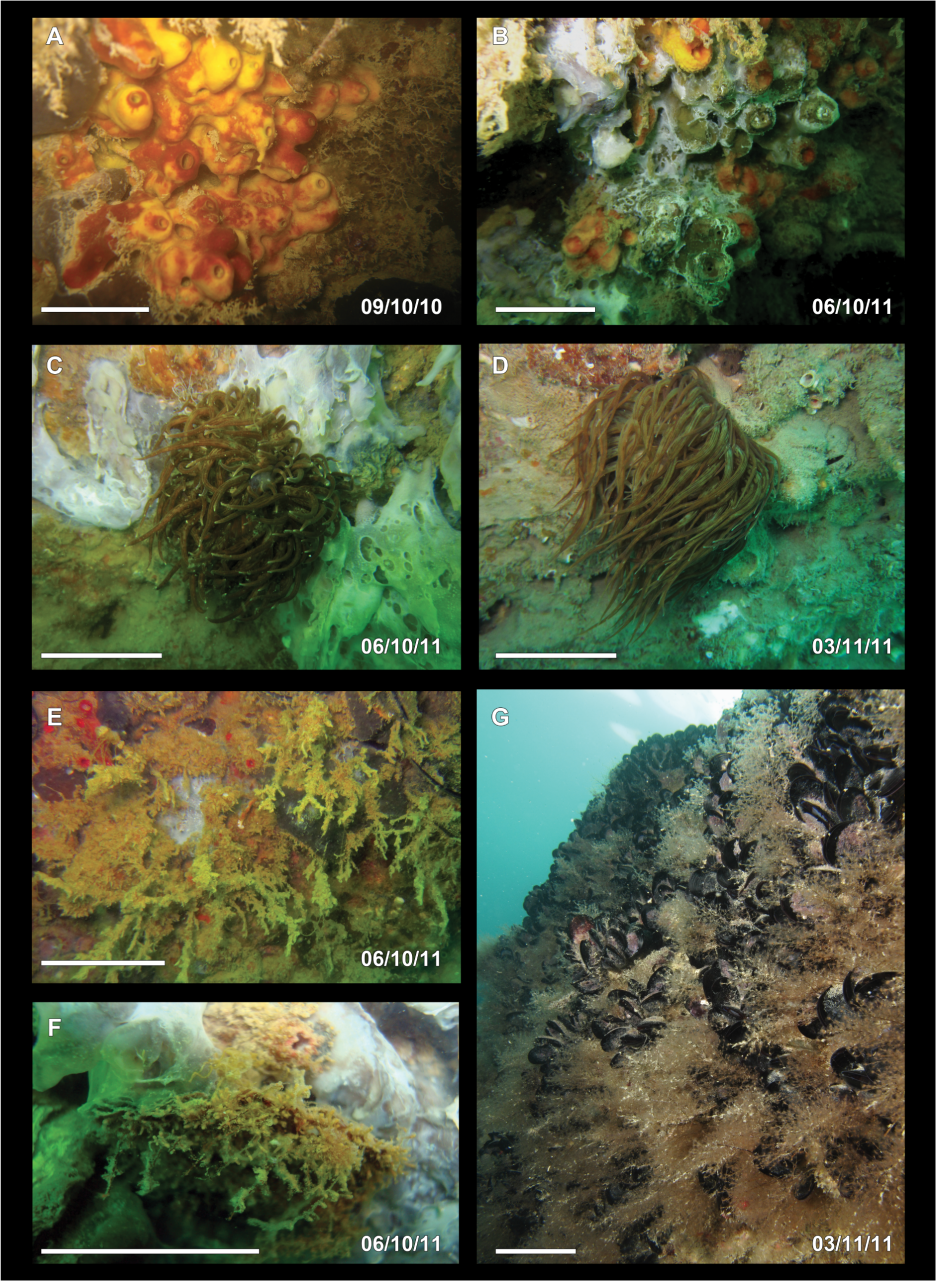
Effects of the disease on some benthic species. A-B) Appearance of *Aplysina aerophoba* before (A) and during (B) the disease. C) The anemone *Aiptasia* sp. was contracted during the disease (C) but it appeared in healthy conditions about one month later (D). E-F) Colonies of *Eudendrium racemosum* lacking of polyps (E) and of *Cornularia cornucopiae* with contracted anthocodia (F). G) Hydroids completely recovered within a month from the disease (picture G by F. Betti). Scale bars A-F 5 cm, G 10 cm.

**Fig 2:**
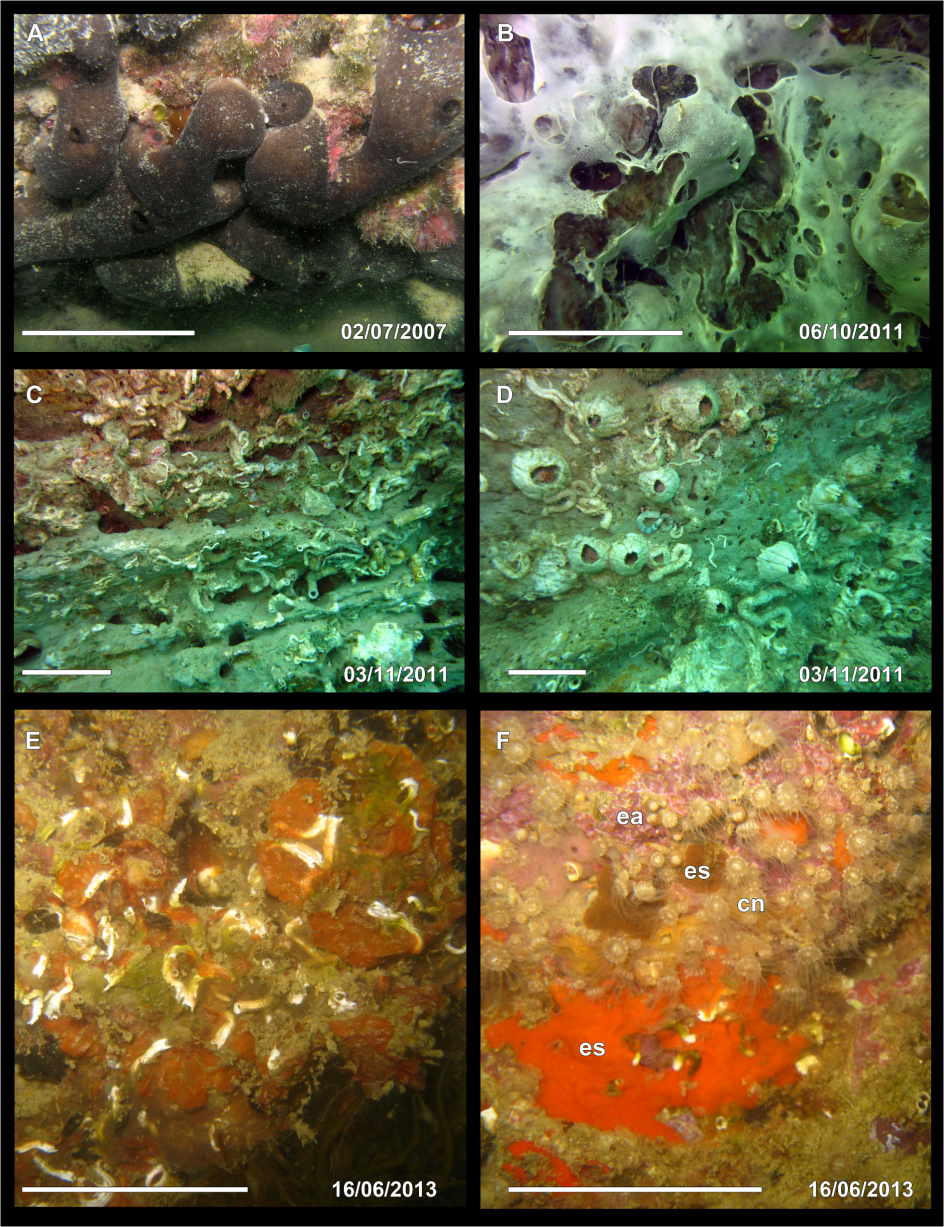
Underwater pictures of different areas occupied by *Chondrosia reniformis* before, during and after the disease. A) Picture of a healthy individual of *C*. *reniformis* taken before the disease. B) During the disease, almost the total surface of *C*. *reniformis* showed necrotic areas characterized by white and putrefied tissues. C-D) Large areas visible in November 2013 after the detachment of dead *C*. *reniformis*; the rocky wall is covered with calcareous tubes of serpulids (C) and cirripeds (D). E-F) Recovery of the area after almost two years; the areas where *C*. *reniformis* was the dominant species (CrAs) were colonized by pioneer species such as the tubeworm *Spirobranchus triqueter* and the bryozoan *Schizobrachiella sanguinea* (E); the areas where *C*. *reniformis* was not the dominant species (OAs) were mainly occupied by cnidarians (cn), encrusting sponges (es) and the encrusting alga *Titanoderma* sp. (ea). Scale bars A-F 5 cm.

The mass mortality event at the Conero Promontory occurred suddenly at the beginning of October, and surveys were performed on the 6^th^ of October 2011. The episode lasted less than a month, since the 3^rd^ of November there were very few diseased organisms. Several species were affected (Figs 1 and 2): some of them, such as the sponges *Ircinia variabilis*, *Sarcotragus spinosulus*, *Spongia officinalis* and *Aplysina aerophoba* were totally or partially damaged (Fig 1A and 1B). The colonies of the cnidarians *Eudendrium racemosum* and *Cornularia cornucopiae* were present but the polyps of *E*. *racemosum* were reabsorbed and those of *C*. *cornucopiae* were contracted (Fig 1E and 1F). Other organisms, such as *Epizoanthus arenaceus*, *Cereus pedunculatus* and *Aiptasia* sp. (Fig 1C) looked as if suffering but they were still reactive. Regarding *Anemonia viridis*, no bleached anemones were observed, but the anemones appeared limp, with contracted and flabby tentacles, dark grey-green in colour always without any violet tips. Some specimens of the polychaete *Sabellaria spallanzanii* were dead, while other species, such as *Dysidea pallescens*, *Haliclona* sp., *Tedania anhelans*, and solitary ascidians were scarcely or not at all hit by any disease. The affected sponges showed wide necrotic areas characterized by white and putrefied tissues (Figs 1B and 2B), while the fur-like coating observed in 2009 on decayed specimens of *S*. *spinosulus* [35], was no recorded any more. The most injuried species was the sponge *Chondrosia reniformis*, with the 100% of the specimens affected (Fig 2A and 2B). In November 2011 all the affected specimens of *C*. *reniformis* disappeared, leaving large bare areas on the rocky and revealing a complex three-dimensional substrate created by large calcareous tubes of serpulids and plates of cirripeds (Fig 2C and 2D). On the contrary, the cnidarians were in good condition, showing extended polyps and healthy colours (Fig 1D and 1G).

### Environmental parameters

**Fig 3:**
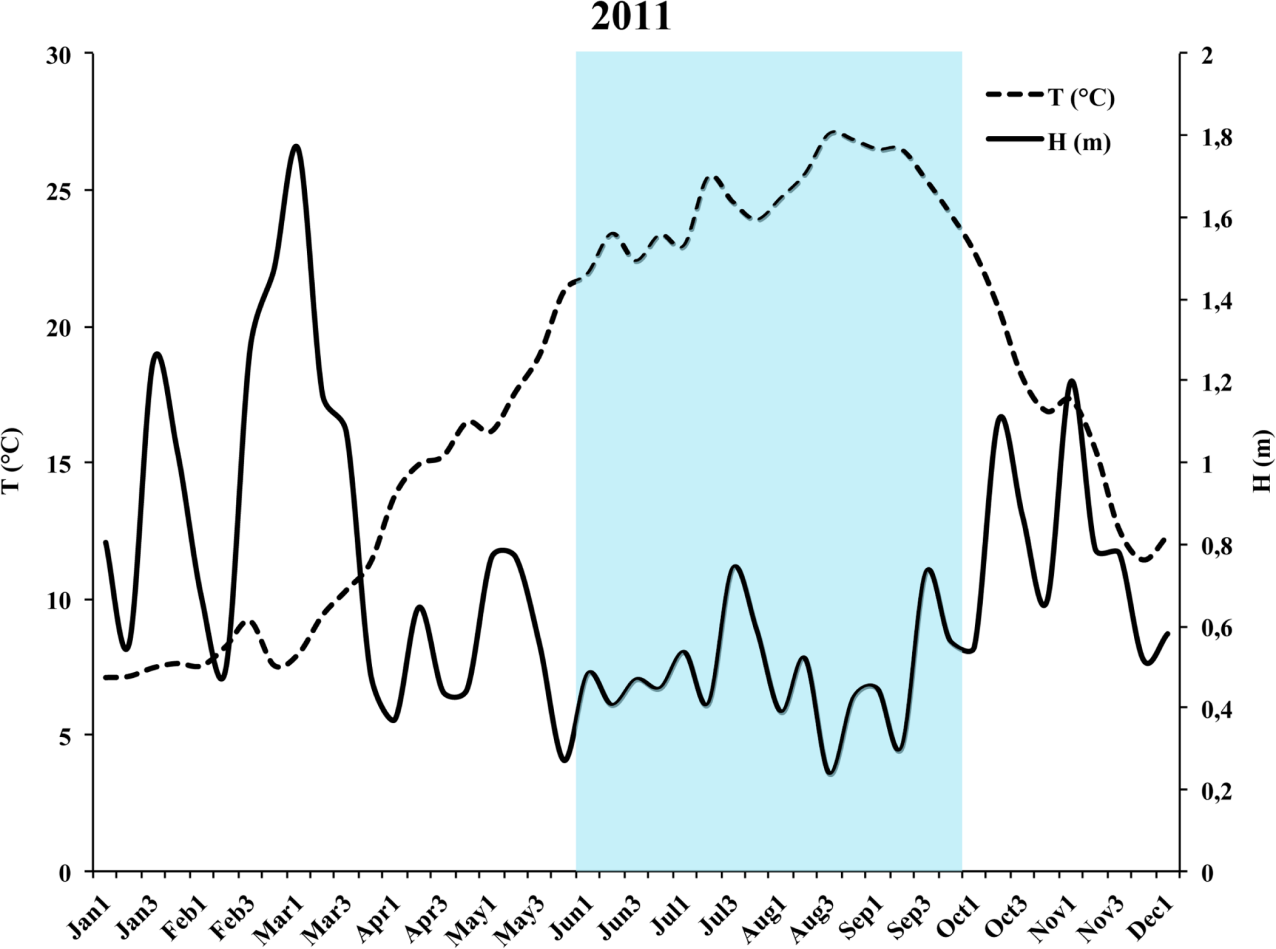
Trend of weekly average values of wave height (m) and superficial temperature (°C) of 2011. The blue area indicates the prolonged period of calm sea conditions and high temperature (from June to the first week of October).

Considering the weekly average values recorded in 2011, the trend of wave height (m) and sea-water temperature (°C) (Fig 3) shows that the disease spread at the end of a prolonged period of a calm sea conditions and high sea-water temperature. From June to the first week of October, the average values of wave height and sea-water temperature were 0.5 m and 24.5°C, respectively. The first day of rough conditions was the 7^th^ of October, but the sea water temperature decreased below 20°C only after the 15^th^ of October.

Data of wave height and sea-water temperature from 2000 to 2013 (Table 3) show that in 2011, the period with calm sea lasted longer than the previous years, while the period with high sea-water temperature was similar to that of the previous years.

**Table 3:** Duration of periods of calm sea and high temperature from 2000 to 2013.

	2000	2001	2002	2003	2004	2005	2006	2007	2008	2009	2010	2011	2012	2013
Max time interval with waves < 0.80 m	6 weeks	12 weeks	7 weeks	**20 weeks**	18 weeks	18 weeks	-	-	-	**15 weeks**	10 weeks	**26 weeks**	-	-
Max time interval with T ≥ 23°C	93 days	77 days	91 days	**99 days**	86 days	-	-	72 days	64 days	**121 days**	77 days	**82 days**	94 days	79 days
Max time interval with T ≥ 27°C	3 days	5 days	3 days	**86 days**	1 day	-	12 days	1 day	2 day	**6 days**	11 days	**5 days**	4 days	5 days
Highest summer temperature (°C)	27.8	28	28.4	**32**	28.4	-	28.4	27.3	27.9	**27.7**	28.2	**27.9**	27.8	27.5

The years characterized by disease were showed in bold.

**Fig 4:**
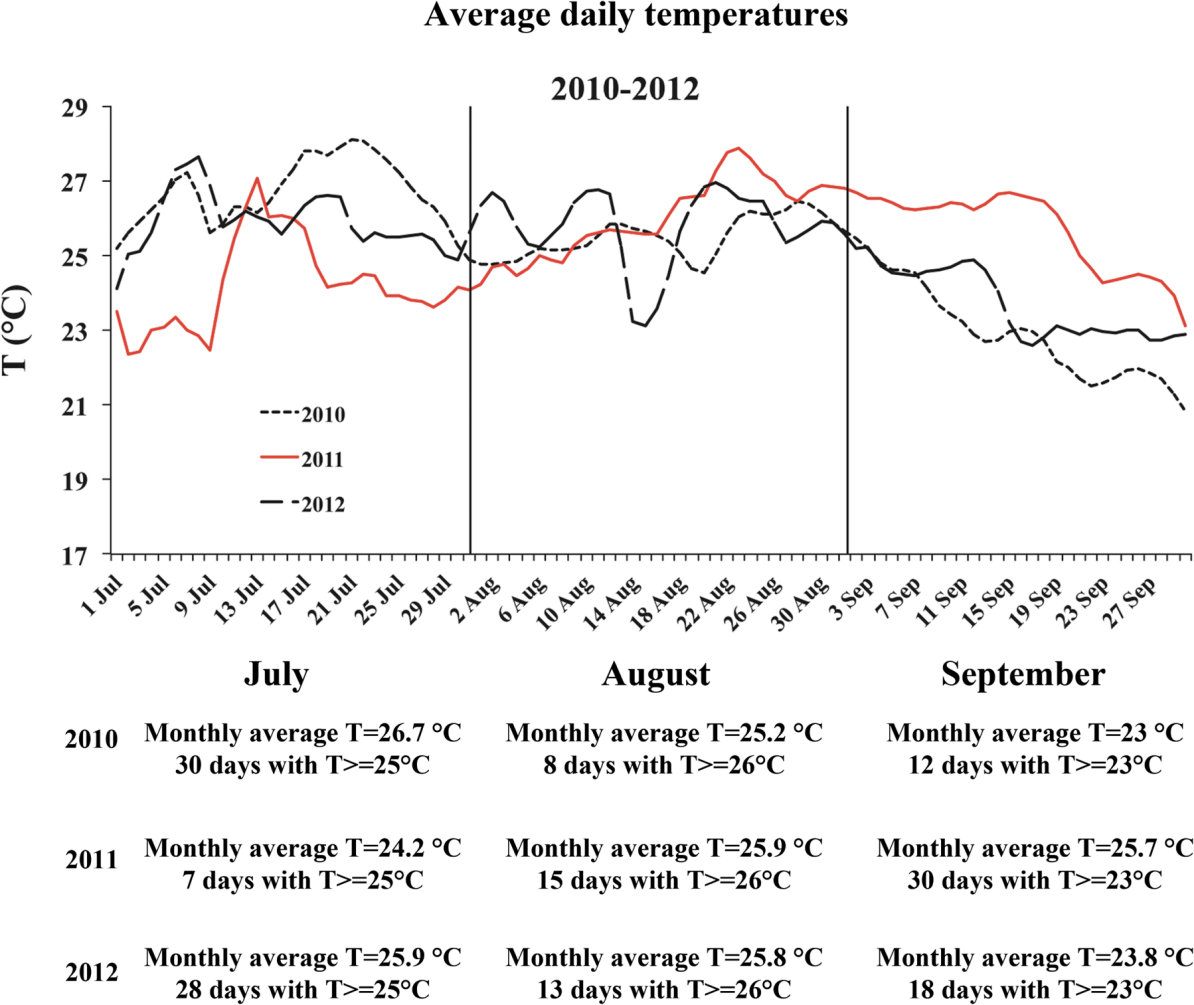
Average daily temperature from July to September of the years 2010–2012. The trend relative to the year with episodes of disease (2011) were showed by a continuous red line. Temperature kept high (≥23°C) throughout September.

The sea-water temperature time series from July to September of the years 2007–2009 [27], and 2010–2012 (Fig 4) show that both in September 2009 and September 2011 temperature persisted over 23°C for 30 days.

In autumn 2011, the conspicuous overflows, favoured by the orientation of the Conero Promontory, changed the usual course of the north-to-south current of the northwestern Adriatic Sea and activated an anticyclonic gyre with a consequent retention of river waters (especially coming from the Po river, located north of Conero Promontory) leading to an impressive algal bloom [43]. During the period September-November, there were no episodes of anoxia/hypoxia [43].

### Analysis of the impacts

Values of abundance of the considered species in 2011 have been indicated in Table 1 together with previously collected data (monthly and annual average values ± SD).

Before the disease, *Epizoanthus arenaceus* from the Conero Promontory showed a yearly average cover of 13.2% ± 8.2 SD while in October 2010 it was 12.5% ± 17 SD [30]. During the disease (October 2011) we observed a 4-fold decrease in the zoanthid cover (2.9% ± 1.7), and most of the polyps were strongly contracted. After the outbreak (November 2011), the colonies recovered, and all polyps were extended and healthy (having a cover of 8.2% ± 9.9).

The abundance of *Cornularia cornucopiae* during the disease (2.2 x 10^3^ of polyps m^-2^ ± 3.5 x 10^3^) was much lower than that recorded before (annual average 12.7 x 10^3^ of polyps m^-2^ ± 8.7 x 10^3^ and 8.6 x 10^3^ ± 0.8 x 10^3^ in October 2009, [36], and all the polyps were reabsorbed or retracted in their calyxes. In November 2011, the species was already in good condition and reached an abundance of 20.3 x 10^3^± 16.6 x 10^3^.

The disease affected only 4% of the *Tedania anhelans* population. The sponge cover was 6.9% ± 3.8 and 2.9% ± 2.5 in October and November 2011, respectively. In both months, the sponge exhibited the typical autumnal encrusting form without the ramified digitations (propagules) by which the sponge asexually reproduces during the spring-summer period [29].

Considering the Irciniidae-Spongiidae complex, the percentage of substrate covered was 19.7% ± 13.2 and 10.5% ± 8 in October and November 2011, respectively. Along the transect 40 m x 1 m, the density of Irciniidae-Spongiidae was 5.7 sponges m^-2^ ± 9.3 and 3.1 sponges m^-2^ ± 9.8. About 70% (160 specimens) of the Irciniidae-Spongiidae complex was in healthy condition, 10% (22) was partially damaged and 20% (46) totally damaged. The analysis of pictures showed that in October, over 56% of the sponge surface showed signs of the disease while in November, the diseased surface was about 9%.

*C*. *reniformis* from the considered area, with specimens larger than 1m^2^ (average percentage of covered substrate 43.2% ± 6.8), was probably one of the largest Mediterranean sponge species [29]. During the disease the sponge cover was 63.1% ± 25.0, and all the sponges were affected with over 98% of the decayed surface. The necrotic areas were white and slimy, and it was easy to detach the necrotic tissues. The cover of *C*. *reniformis* drastically decreased to 6.3% ± 4.9 immediately after the disease (November 2011).

Colonies of *E*. *racemosum* are generally abundant on the rocky wall of Scoglio del Trave from spring to early autumn [28]. During the disease, the hydroid colonies were present but almost completely lacking of polyps. On the 3^rd^ of November we observed fully recovered colonies with 34.1 hydranths cm^-2^ ± 4.8 (Table 1).

### Recovery

**Fig 5:**
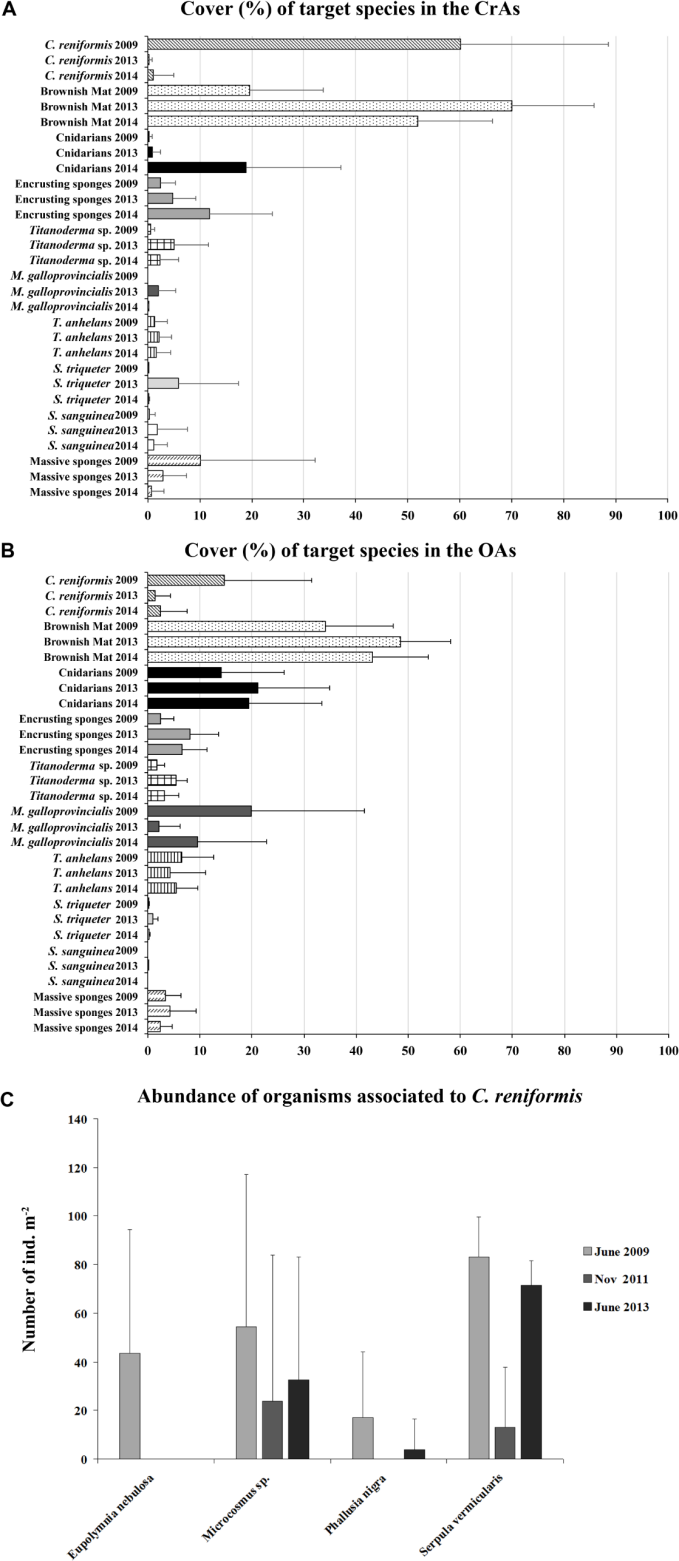
Recovery of the study area. The bar charts show the comparison of the abundance of the most representative species before (June 2009) and after the disease (June 2013 and 2014) both in the areas where *C*. *reniformis* was the dominant species (CrAs) (A) and in the other areas (OAs) (B). C) Abundance of fauna associated to *C*. *reniformis* before (June 2009), immediately after (November 2011) and almost two years after the disease (June 2013).

Considering the areas dominated by *C*. *reniformis* (CrAs), the sponge cover was 60.2% ± 19.6 SD in 2009 and 0.22% ± 0.6 SD in 2013 (Fig 5). In 2013 the cover of the massive sponges in the CrAs diminished, while the bare areas once occupied by *C*. *reniformis* were colonized by pioneer species such as the serpulid *Spirobranchus triqueter* and the bryozoan *Schizobrachiella sanguinea* and the organisms of the BM (about 70.0% ± 15.8). Considering the other areas (OAs) before the disease, *C*. *reniformis* covered an area 14.7% ± 16.8. The rest of the substrate was colonized by a mosaic of invertebrates such as cnidarians (14.1% ± 12.1), several encrusting sponges (2.7% ± 2.5), *M*. *galloprovincialis* (19.8% ± 21.7) and a red encrusting alga (*Titanoderma* sp., 1.8% ± 1.5). In 2013, *C*. *reniformis* severely diminished even in the OAs with an average cover of 1.4% ± 3. The bare areas previously occupied by *C*. *reniformis* were mainly colonized by the surrounding organisms and in particular by cnidarians (21.3% ± 13.2), encrusting sponges (7.8% ± 5.5) and *Titanoderma* sp. (5.3% ± 2.2). The cover of *M*. *galloprovincialis* diminished to 2.1% ± 4 while the BM increased to 48.2% ± 9.3.

In 2014, the BM of the CrAs diminished to 51.9% ± 15.4, while encrusting sponges and cnidarians, mainly represented by the hydroid *Obelia dichotoma*, increased (11.8% ± 12.1 and 19% ± 18.2, respectively). In the OAs less evident changes occurred. The percentage of cnidarians was 19.4% ± 14 while the cover of *M*. *galloprovincialis* slightly increased to 9.5% ± 13.2.

Between 2009 and 2013, significant variations in the cover of *C*. *reniformis* (both in the CrAs and OAs: Kruskal-Wallis, H = 20.6; p<0.001 and H = 3.7; p<0.05), encrusting sponges (only in the OAs: H = 6.8; p<0.01), *S*. *triqueter* (both in the CrAs and OAs: H = 18.1; p<0.001 and H = 14.3; p<0.001), *M*. *galloprovincialis* (both in the CrAs and OAs: H = 3.7; p<0.01 and H = 6.1; p<0.05), *Titanoderma* sp. (both in the CrAs and OAs: H = 5.3; p<0.05 and H = 12.3; p<0.001) and brownish mat (both in the CrAs and OAs: H = 19.1; p<0.001 and H = 8.1; p<0.01) were recorded. Between 2013 and 2014, significant variations occurred in the cover of cnidarians (p<0.001 and H = 15.4), *S*. *triqueter* (p<0.001 and H = 16.5) and BM (p<0.01 and H = 9.3) in the CrAs.

Concerning the fauna associated to *C*. *reniformis* (Fig 5C), the abundance of the considered taxa diminished after the disease (November 2011), and in June 2013 the values were still lower than June 2009. In particular, the polychaete *Eupolymnia nebulosa* was observed neither in November nor in June 2013.

**Fig 6:**
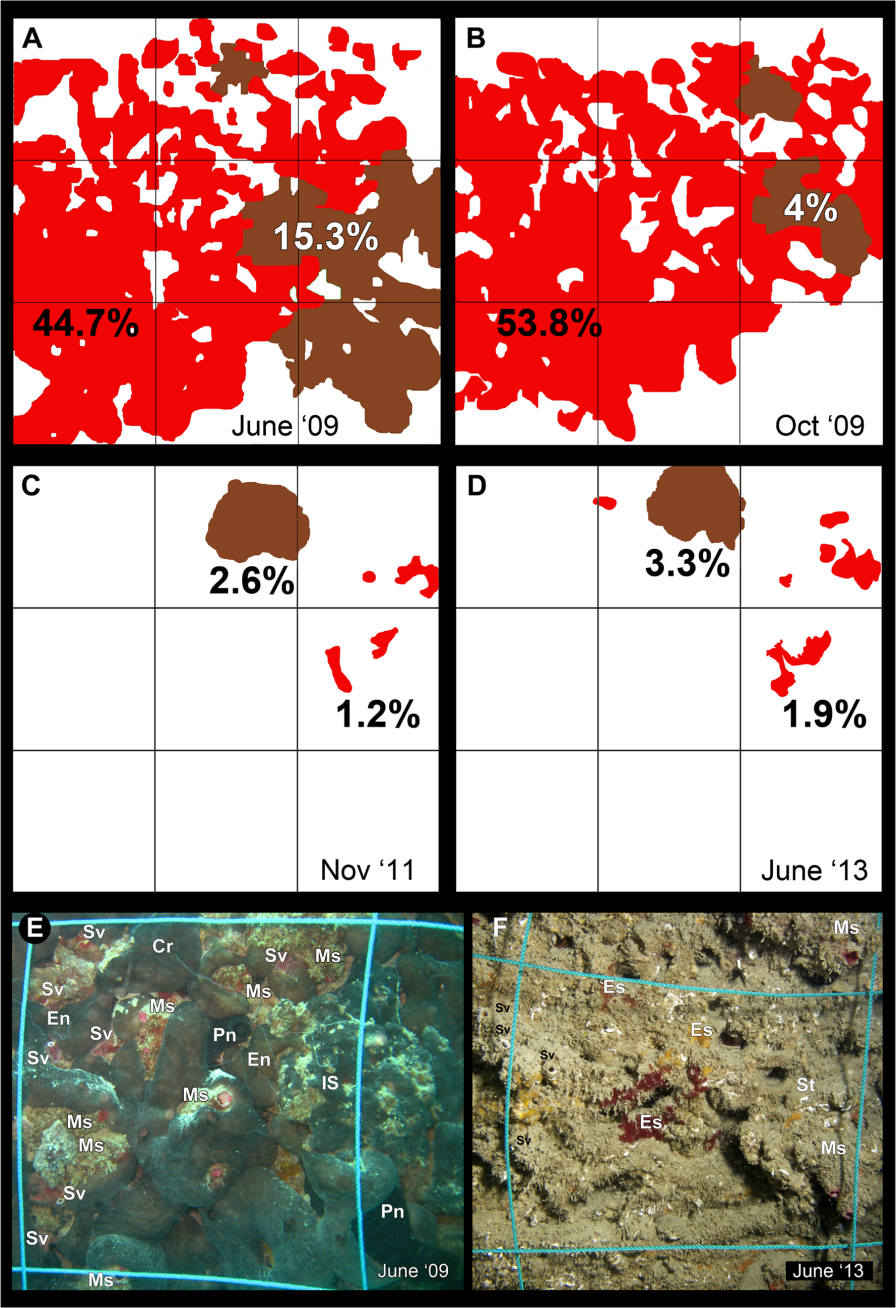
Reduction of *Chondrosia reniformis* and Irciniidae/Spongiidae communities after the two disease events. Scheme illustrating a 0.5 x 0.5 m marked area subdivided in 9 sub-quadrats of 16.7 cm side in four different moments: before (June 2009) (A) and after (B) (October 2009) the first outbreak and after the second outbreak (November 2011 (C) and June 2013 (D)). The surfaces covered with *Chondrosia reniformis* and Irciniidae/Spongiidae were indicated in red and brown, respectively; numbers indicate the total cover of each species. Pictures E-F shows the central area of the quadrat (16.7 cm side) colonized by different assemblages in June 2009 (D) and June 2013 (D). Cr: *Chondrosia reniformis*, En: *Eupolymnia nebulosa*, Es: Encrusting sponges, Ms: *Microcosmus* sp., Pn: *Phallusia nigra*, IS: Irciniidae/Spongiidae, St: *Spirobranchus triqueter*, Sv: *Serpula vermicularis*. Figure F by F. Betti.

The scheme in Fig 6 shows the comparison of the marked area in four different moments: before (Fig 6A and 6E) and after the disease of 2009 (Fig 6B) and after the outbreak of 2011 (Fig 6C, 6D and 6F). *C*. *reniformis* was not affected at all by the disease of 2009, while the surface occupied by Irciniidae-Spongiidae resulted drastically reduced (from about 15% to 4%). In November 2011, both *C*. *reniformis* and Irciniidae-Spongiidae showed a decrease in the sponge cover (Fig 6C). The *C*. *reniformis* specimens which survived the second outbreak grew to about 16 cm^2^ in 19 months (from November 2011 to June 2013, Fig 6D), with an estimated growth rate of about 0.03 month^-1^.

Table 2 shows that the total volume filtered by 6 species living in an area of 6000 m^2^ decreased from about 160 10^6^ L h^-1^ to 52 10^6^ L h^-1^ from 2009 to 2013, with a loss of the filtration activity by over 60%. In particular, the species with the highest filtration activity are the ascidians *Microcosmus* spp. (68 10^6^, about 42% of the total) and the sponge *Chondrosia reniformis* (60 10^6^; about 37%), but their contribution to the total filtration efficiency diminished by 40% and 97%, respectively.

## Discussion

### Overview and possible causes of the outbreak

From 2007 to 2011—two strong disease outbreaks were reported from the Conero Promontory at the end of summers 2009 and 2011. The last episode occurred during a period with the highest average surface sea-water temperature recorded in Italy during the 30-year time series from 1961 to 1990 [44], which affected a higher number of species. The long-lasting water warming occurred in summer/early autumn concomitantly with the absence of rainfalls, and the eutrophic waters coming from the northern rivers most likely triggered the spreading of the disease. Since hypoxic events never occurred throughout 2009 and 2011, the causes of the mortality remain unclear. Recently, several Authors [13, 15–16] affirmed that pathogenic bacteria of the thermophilic genus *Vibrio* could trigger mass mortality episodes. In the two recent mortality events, which occurred at the Conero Promontory, different species of sponges were involved and, in 2011, even species belonging to other phyla were affected. The weather conditions in 2011 were not so extreme in order to justify severe mass mortality, and other factors, probably of anthropic origin, controlled the proliferation of pathogens and the susceptibility of the affected organisms. On 22 August 2011, the ship Artiglio AN 4067 CARMAR discharged polluted muds along the coast at a distance of less than 7 km north of Scoglio del Trave [45]. It is not possible to have data directly supporting a cause-effect relationship but we can speculate that the pollutants combined with critical weather conditions, might have contributed to this dramatic disease.

In spite of the fact that the study area was considered a land-based pollution hot spot [46], the consequences of forms of impact different from fisheries (maritime traffic, aquaculture, oil and gas drilling, pollution, tourism, beach nourishment) on benthic assemblages are poorly explored [47–48], especially concerning their role in triggering or enhancing mass mortality events.

### Direct effects of the outbreak

The outbreak of 2011 caused evident shifts in the benthic assemblage of the study area. In particular, the slow-growing species were the most affected during the disease. From the 7^th^ to 31^st^ of October, at least 6 episodes of storms were recorded along the coast with waves of up to 2.7 m (ISPRA data). The abundance of the massive sponges was seen to have diminished in November 2011 likely due to the storms which caused the detachment of the whole dead specimens or of the necrotic portions [35].

The common sponge *T*. *anhelans* did not show any sign of disease in 2009 and was scarcely damaged in 2011 suggesting that it is a very tolerant species. The variations of the cover observed during the surveys were probably due to the natural shrinkage/expansion phases typical of the sponge life cycle [29].

On the contrary, the large sponges specimens of *C*. *reniformis* almost disappeared from the study area (S1 Video). In the Mediterranean, this species was never affected in previous mortality events. Some Mediterranean sponges have relatively slow growing dynamics [41]. Our surveys have confirmed that *C*. *reniformis* is a conservative sponge and suggest that the species will employ a long time to come back to its original abundance and dimensions. The estimated growth rate of the surviving fragments of *C*. *reniformis* in the marked area is 0.03 month^-1^. This evaluation does not consider the larval settlement and the fusion of ramets [49], as well as other factors—seasonal fluctuations of environmental parameters, interaction of other organisms, food availability [41, 50–51]—which could affect the growth pattern. In any case, the growing process in Demospongiae is slow [42, 52], suggesting that other metazoans more resistant to future outbreaks or those that are able to recover quickly could replace the slow-growing sponges. The disease of *C*. *reniformis* also affected on the neighboring organisms. When rough conditions detached dead specimens of *C*. *reniformis*, many of the animals living in association with the sponges were extirpated together. Hence, *C*. *reniformis* probably camouflaged the associated fauna and protected the organisms against storms, acting as a cushion. The bare areas left after the sponge detachment were initially (2013) colonized by fast growing species such as the polychaete *Spirobranchus triqueter* and the bryozoan *Schizobrachiella sanguinea*. Moreover, the organisms of the BM, mainly composed of cnidarians, triplicated their cover in the CrAs. These pioneer species quickly colonize new available areas, especially in the absence of sciaphilous algae [53–55]. These organisms are successful mainly due to their high growth rates. *S*. *triqueter* can grow fifty-fold faster [56] than *C*. *reniformis*. Moreover, bryozoans can produce antibacterial compounds preventing the development of a microbial biofilm [57] indispensable for larval settlement [58]. Pioneer hydroids can use their cnidocysts to anchor on virgin substrates [59] or discourage settlement of other organisms [53–60]. Hydroid predation on merobenthic larvae [28] may also impede or delay larval settlement [60] (Standing 1976) of other species.

In 2014, the pioneer species were replaced by cnidarians and encrusting organisms (algae, sponges and ascidians). Before the disease, the cold-affinity hydroid *Obelia dichotoma* formed few tufts in a restricted zone in the CrAs, while in June 2014, the hydroid constituted a belt covering a much larger area. In the OAs, cnidarians and mussels were among the most resilient organisms.

### Possible long-term ecological consequences

The macrozoobenthos of hard substrates influences water properties and nutrient cycling and provides several ecosystem services playing a pivotal role in the benthic pelagic coupling processes, retention of organic matter and carbon sequestration [61]. The loss of benthic organisms may have a cascade effects on the neighboring species and may cause the alteration of the functioning of the ecosystem [62].

After the disease of 2009, the filtration activity of the study area decreased by about 5%, while after the second outbreak, the loss was over 60%. Consequent effects on the area are unpredictable. Moreover, the influence of *C*. *reniformis* on silica turnover [63] (Cerrano et al. 1999) may also affect primary productivity at a small local scale. A classic case study is known from Chesapeake Bay (Western Atlantic) where, before the 1870s, oyster populations from Chesapeake Bay (Western Atlantic) were able to filter all the water of the estuary in less than 1 week [64]. In the 19^th^ century, massive overfishing and mechanical destruction of oyster beds reduced the filtration activity of the stocks by 50-fold with a consequent increase in episodes of eutrophication and hypoxia [64]. Sponges can establish symbiotic relationships with bacteria, and more than 50 percent of the wet weight of a sponge can be composed of prokaryotes [65]. Through their filter feeding activity, sponges remove nano- and pico-phytoplankton, dissolved organic carbon and viruses from the water column playing a role in the microbial loop [66–67]. Due to their involvement in bentho-pelagic coupling [67], and their ability to modify, maintain and/or create habitats [68], sponges can be considered as ecosystem engineers. The depletion of the large and abundant sponge *C*. *reniformis* and its associated organisms in the studied area could lead to a simplification of the benthic ecosystem [69].

Considering documented regime shifts all over the world, temperature stress and anthropogenic pressures can lead to dramatic changes in benthic communities [70–71]. In temperate waters, the shift from highly productive algal forests to ‘barrens’ was observed [72–74], such as the loss of anthozoan forests [6, 75] leading to a regime shift from crustose coralline to green filamentous algae.

In coralline reefs, the most common scenario is the transition from hard corals to macro- or turf algae [76] communities. However, there is some evidence of shifts from a coral- to sponge-dominated system in several localities (Caribbean, Atlantic, Indo-Pacific and Pacific reefs, [77] and references therein). At Santos Bay (Brazil), there is a ‘coral reef crisis’ at present: a phase shift, in which reef-building corals are replaced by the soft-bodied anthozoan *Epizoanthus gabrieli* [78]. In coral reefs, water warming, several anthropogenic impacts, and in particular ocean acidification, can compromise the biomineralization of hard corals and polyp activity [79] and induce shifts from hard coral to other communities [80–81].

Concerning the North Adriatic Sea, we witnessed the transition from a massive sponge dominated habitat to a mosaic of fast-growing species with a plastic life cycle, where cnidarians prevailed. The high resilience of soft-bodied cnidarians has already been documented for the basin: the sea anemone *Cereus pedunculatus* was the only surviving sessile organism after experimentally inducing small-scale anoxia in the north Adriatic Sea [82]. Sea anemones are highly resistant to mass mortality events [83], and in experiments of induced oxygen crisis they exhibit particular behaviours such as predation on the species that are more vulnerable to anoxic conditions [84].

A sponge-cnidarian phase shift could entail a passage from filter feeders, collecting particles not exceeding 20 μm, to carnivorous, which in this area, feed especially on merobenthic larvae [28]. On a small scale, this change in the functional groups could influence nutrient cycling and lead to a decrease in the zooplankton and an increase in bacteria and phytoplanktonic organisms.

The NW Adriatic Sea is prone to mass mortality especially in late summer both for the physical characteristics of the basin [18, 35, 82–85] and for cumulative human impacts [86]. Considering the frequency of extreme climatic events is expected to increase [48], the loss of a consistent biomass of filter feeders will enhance the risk of triggering new eutrophic phenomena and mass mortality episodes [87]. A continuously degraded habitat has less chance to recover from multiple disturbances. On the contrary, preserved areas show major ability to resist to diseases due to the integrity of the habitat and high diversity [88]. Long-time monitoring activities of the study area are needed to observe the succession of the organisms and forecast the rates of change of the communities [89].

## Conclusions

In the North Adriatic Sea, cnidarians are one of the major components of the benthic assemblages, and they are more resistant and resilient than massive sponges. Long-term consequences of environmental stresses could likely promote the replacement of slow-growing sponges in favour of more resilient organisms. The Mediterranean Sea can be considered as a miniature ocean which can serve as a natural mesocosm of the oceans [3]. Long term studies at regional scale are crucial to monitor changes of the Mediterranean biota [90–91]. Because of the reduced dimension of the basin, the North Adriatic Sea responds more quickly to climatic anomalies and other environmental stresses and it should be regarded *a priori* as an ecosystem that is more sensitive to fluctuations of physical parameters [85]. The present study, conducted in the North Adriatic Sea, provides the baseline for future long term studies on expansions and retractions of benthic species from an area considered of strategic importance to assess the effects of global change and which should be monitored with special care [90, 92].

## Supporting Information

S1 Video Video showing the bare wall once occupied by *C*. *reniformis* (Video taken in August 2014). (MP4)
